# The Expression of Mucin-4 (MUC4) in Sarcomas Apart From Sclerosing Epithelioid Fibrosarcoma and Low-Grade Fibromyxoid Sarcoma

**DOI:** 10.7759/cureus.49546

**Published:** 2023-11-28

**Authors:** Usman Hassan, Saad M Saeed, Sajid Mushtaq, Mudassar Hussain, Maryam Hameed

**Affiliations:** 1 Pathology, Shaukat Khanum Memorial Cancer Hospital and Research Centre, Lahore, PAK

**Keywords:** sef, lgfms, synovial sarcoma, sclerosing epithelioid fibrosarcoma, low-grade fibromyxoid sarcoma, muc4

## Abstract

Background

Low-grade fibromyxoid sarcoma (LGFMS) and sclerosing epithelioid fibrosarcoma (SEF) are two rare but aggressive soft tissue sarcomas that can be difficult to distinguish due to histopathological similarities. The present study examines the diagnostic capacities of mucin-4 (MUC4), a transmembrane mucin, in identifying different types of sarcomas and broadens its evaluation to include a wide range of sarcomas.

Methods

Immunohistochemical (IHC) examination of tissue samples from various sarcomas was performed using a mouse anti-MUC4 monoclonal antibody. IHC was conducted on 4-mm thick formalin-fixed paraffin-embedded tissue sections after pressure cooker antigen retrieval with a mouse anti-MUC4 monoclonal antibody.

Results

MUC4 was shown to be highly expressed in SEF (n=13) and LGFMS (n=10), while focal positivity in synovial sarcoma (n=1). Other sarcomas, such as malignant peripheral nerve sheath tumors, fibrosarcoma, leiomyosarcoma, liposarcoma, and myxofibrosarcoma, exhibited no expression (n=0). These findings are consistent with previous research and support MUC4 specificity as a SEF and LGFMS marker. This study provides information on the diagnostic efficacy of MUC4, particularly in the context of certain subtypes. It not only helps our understanding of these unique instances, but it also provides context for histopathological and IHC findings in soft tissue sarcoma. Furthermore, this study investigates the influence of age and gender on MUC4 expression in a range of sarcomas, which was typically understudied in the literature and found no relation with expression of MUC4.

Conclusion

In conclusion, this study adds to our understanding of soft tissue sarcomas by emphasizing the crucial role of MUC4 in certain sarcoma subtypes while acknowledging the complex variety of the sarcoma landscape. Further research is needed to understand the molecular mechanism that governs marker expression patterns, as well as the therapeutic implications.

## Introduction

Sclerosing epithelioid fibrosarcoma (SEF) is an uncommon yet aggressive variety of soft tissue sarcoma that mostly affects young people. At the microscopic level, SEF has a specific histopathological profile highlighted by the close clustering of epithelioid cells inside a fibrosarcomatous stroma. Due to its remarkable similarities to other sarcomas and benign tumors, its unusual cellular arrangements present a difficult diagnostic challenge. Therefore, there is a significant need to find accurate immunohistochemistry (IHC) markers that can clearly identify SEF from its histological imitators [1]. The presence of a fibromyxoid matrix and a variety of cellular characteristics describe low-grade fibromyxoid sarcoma (LGFMS), which presents a similar difficult diagnostic situation [2-5]. It is important to develop and use reliable diagnostics methods that can distinguish this particular sarcoma subtype since the histological appearance of LGFMS frequently resembles that of diverse soft tissue cancers.

Soft tissue sarcomas are difficult to diagnose due to their rarity, rapidly evolving histopathologic and molecular diagnostic classification, overlapping morphology and immunophenotype with many other neoplasms, and reliance on ancillary IHC and molecular investigations for many diagnoses. In the general population, the incidence of malignant soft tissues is estimated to be 25-50 per million [6,7].

Mucin-4 (MUC4), a transmembrane mucin, is critical in epithelial renewal and differentiation. Recent research indicates that MUC4 is involved in pancreatic cancer etiology and is expressed in a variety of normal and malignant tissues. The underlying characteristics of MUC4 in diverse cancer types may enable us to provide suitable treatment and patient monitoring. However, MUC4 contributions to pan-cancer have not been well studied [8].

MUC4 has been reported as a potential diagnostic marker for both SEF and LGFMS in several robust investigations that have been published in multiple studies [4,5]. Studies have shown the great sensitivity of MUC4 as well as its critical use in properly detecting these difficult malignancies. However, there are still many unsolved problems regarding the sensitivity and specificity of MUC4 as a diagnostic marker across a broad spectrum of sarcoma subtypes in the larger context of sarcoma pathology. These concerns are still stimulating current investigations aimed at elucidating the complexities of MUC4 diagnostic potential. Our explorations of MUC4 activity in the context of sarcoma diagnosis go beyond SEF and LGFMS. This study investigates the intricate intricacies of sarcoma pathology, examining the potential diagnostics sensitivity of MUC4 in not just these two particular subtypes but also in a wide range of sarcomas. This study was carefully designed to use “mouse anti-MUC4 monoclonal antibody” as the cornerstone of our research methods in order to achieve objectives.

The choice of the “mouse anti-MUC4 monoclonal antibody” is a critical component of our study’s design. Previous studies have shown that this particular antibody is a very promising technique for identifying MUC4 expression in a variety of biological situations [5]. It stands out because it is monoclonal, which guarantees a great level of uniformity and reproducibility qualities crucial in a study of this scope and significance. Additionally, the deliberate application of an antibody directed against MUC4 in a mouse model complies well with widely accepted IHC criteria [4,6,8]. This method, which has long been praised for its accuracy and dependability, enables us to understand the complex expression patterns of certain proteins inside tissue samples. This study investigated the MUC4 expression by utilizing IHC in SEF and other epithelioid tissues to determine its potential diagnostic utility.

## Materials and methods

Formalin-fixed and paraffin-preserved tissue blocks of already confirmed cases were retrieved from the surgical pathology and consultation files of Shaukat Khanum Memorial Cancer Hospital and Research Center, Lahore, Pakistan. Representative hematoxylin and eosin-stained slides were reviewed. In total, whole-tissue sections of 132 tumors were evaluated for expression of MUC4: 15 cases of leiomyosarcoma, 10 cases of malignant peripheral nerve sheath tumor (MPNST), 23 cases of liposarcoma, 16 cases of SEF, 14 cases of LGFS, 26 cases of myxofibrosarcoma, 15 fibrosarcoma and 13 cases of synovial sarcoma.

IHC staining of various antibodies including MUC4, S100 protein, MDM2, p16, Desmin, Caldesmon, and TLE1 was studied. IHC was conducted on 4-mm thick formalin-fixed paraffin-embedded tissue sections after pressure cooker antigen retrieval (0.01 M citrate buffer, pH 6.0) for all the anti-bodies including a mouse anti-MUC4 (8G7) monoclonal antibody (Cell Marque Corporation, Rocklin, CA), 1 ml concentrate of TLE1 (1F5) mouse monoclonal antibody (Cell Marque Corporation, Rocklin, CA), mouse monoclonal (2A10) anti-MDM2 antibody (Abcam, Boston, MA), mouse monoclonal anti-p16 (E6H4) antibody (Roche Diagnostics, Basel, CH), rabbit anti-s100 polyclonal antibody (Roche Diagnostics, Basel, CH), mouse anti-Desmin (DE-R-11) antibody (Roche Diagnostics, Basel, CH) and Caldesmon (E89) rabbit monoclonal antibody (Roche Diagnostics, Basel, CH). Throughout the study, appropriate positive and negative controls were applied. Positive controls include normal colonic epithelium for MUC4, normal blood vessels lined by endothelial cells for TLE1, de-differentiated liposarcoma for MDM2, normal pancreatic tissue for p16, melanoma tissue for S-100 protein, normal smooth muscle of intestinal tissue for desmin and uterine smooth muscle for caldesmon. A panel of pathologists reviewed the slides using an advanced microscope (Olympus CX23, Olympus Corporation, Tokyo, JPN). MUC4 expression was graded as weak, moderate, and strong based on the intensity of positively stained cells. While expression of other antibodies i.e. TLE1, MDM2, p16, S-100 protein, Desmin, and Caldesmon was evaluated as positive or negative for the corresponding tumors.

## Results

**Figure 1:**
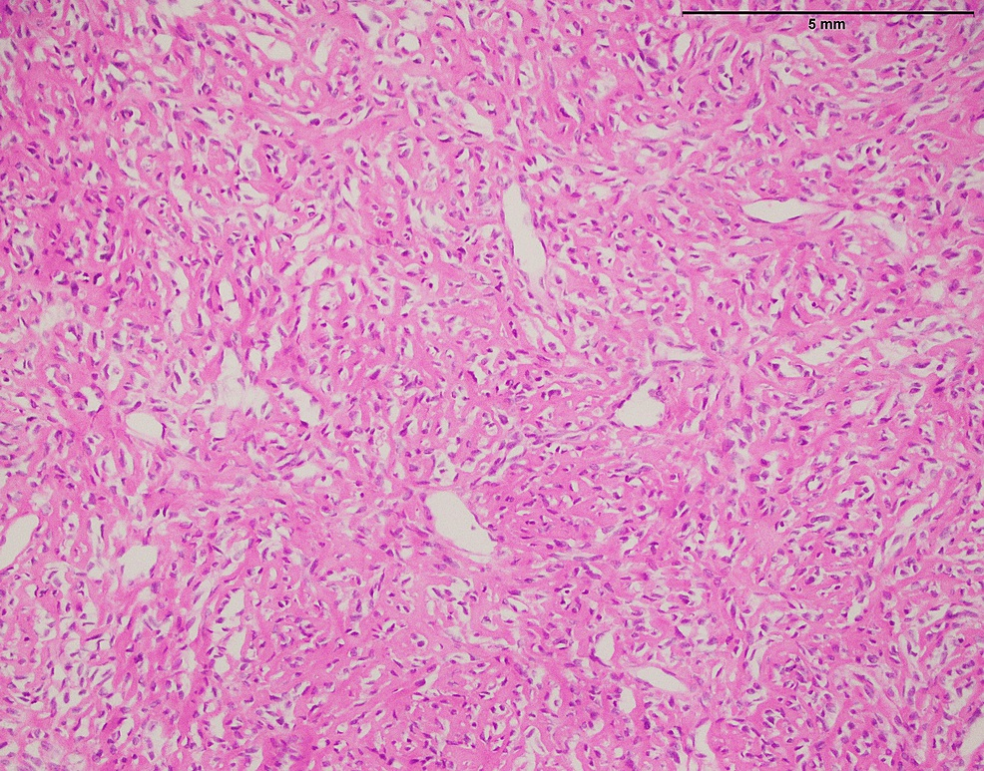
Histopathological characteristics of fibrosarcoma involving the thigh; an infiltrative tumor composed of spindle cells with moderate to marked atypia (40x)

The 15 fibrosarcoma cases affected ten males and five females. The age ranges from 23 to 77 years with mean and median ages of 41 and 42, respectively. The most common anatomical site was the lower limb (n=8) followed by the upper limb (n=6), and abdominal wall (n=1). The characteristics infiltrative growth pattern (Figure 1) indicate that it tends to infiltrate surrounding tissues and structures. This infiltrative nature is a feature of fibrosarcoma, making them particularly difficult to remove entirely after surgery and contributing to their aggressive behavior. Within the tissue, spindle cells were also seen. These spindle cells are elongated and have a spindle-like shape, which is a distinctive feature of fibrosarcoma. Importantly, these spindle cells showed a variety of abnormalities, including moderate to severe atypia (Figure 1). Atypia ranging from mild to severe indicates a major divergence from normal cellular morphology and high-level malignancy.

**Figure 2:**
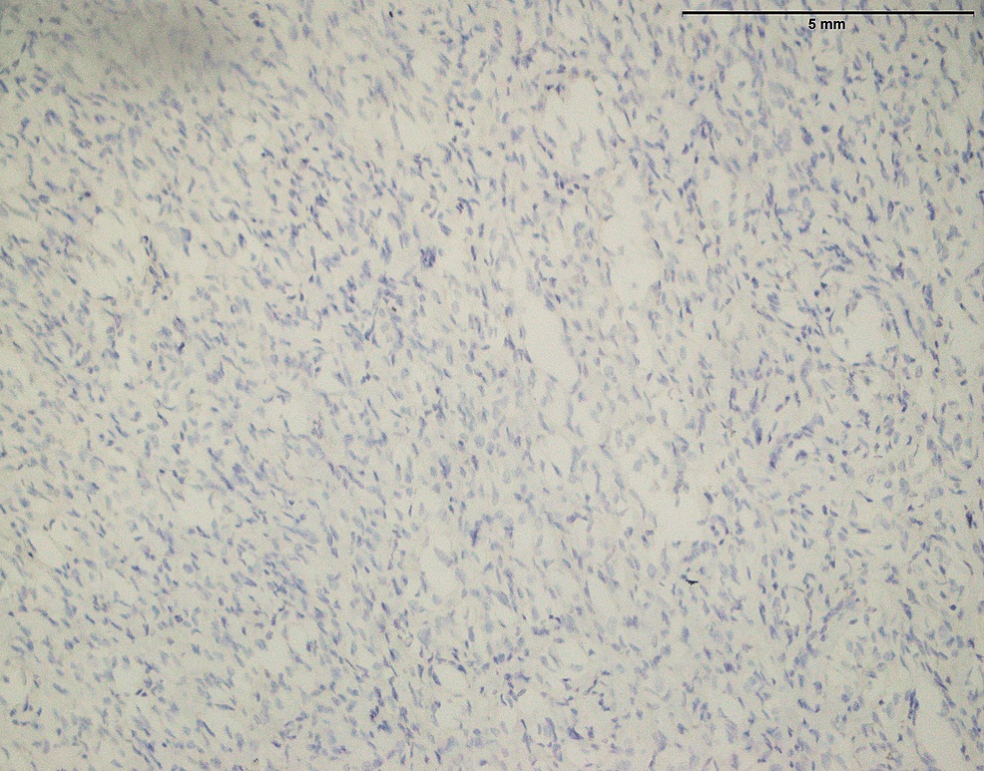
Fibrosarcoma involving thigh: No expression of MUC4 (40x) MUC4: Mucin-4

Figure 2 of fibrosarcoma showed no MUC4 expression. This suggested that MUC4 protein was absent within tumor cells (Table 1). MUC4 is frequently linked to cancer and is known to contribute to development and aggressiveness. In this case, the absence of MUC4 expression may indicate a distinct molecular profile or mechanism at work in the fibrosarcoma, which might have consequences for prognosis and therapy. More research is needed to determine the implications of this negative MUC4 expression in fibrosarcoma.

**Table 1:** Expression of MUC4 in various sarcomas MUC4: Mucin-4

Serial Number	Type of Tumor	Total Cases	MUC4 Positive (%)
1	Leiomyosarcoma	15	0 (0)
2	Malignant peripheral nerve sheath tumor	10	0 (0)
3	Liposarcoma	23	0 (0)
4	Myxofibrosarcoma	26	0 (0)
5	Fibrosarcoma	15	0 (0)
6	Synovial sarcoma	13	1 (8)
7	Sclerosing epithelioid fibrosarcoma	16	13 (81)
8	Low-grade fibromyxoid sarcoma	14	10 (71)

**Figure 3:**
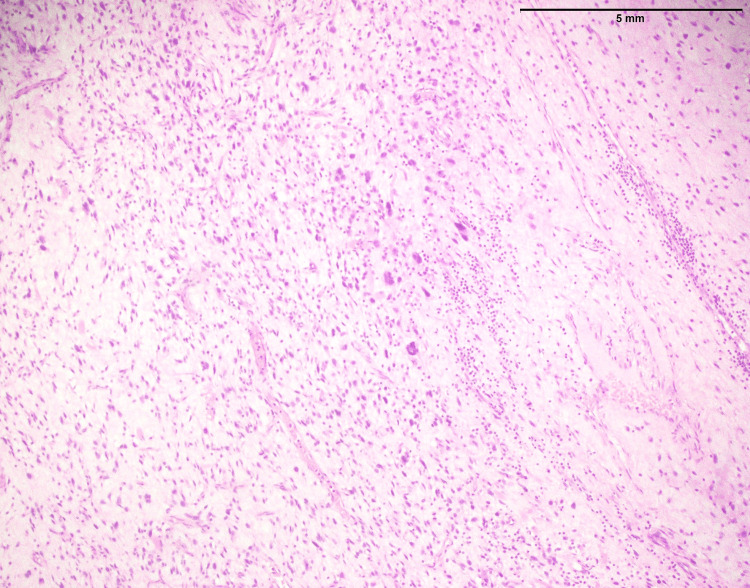
Liposarcoma involving retroperitoneum, showing myxoid background composed of sheets of lipoblasts and giant cells (20x)

**Figure 4:**
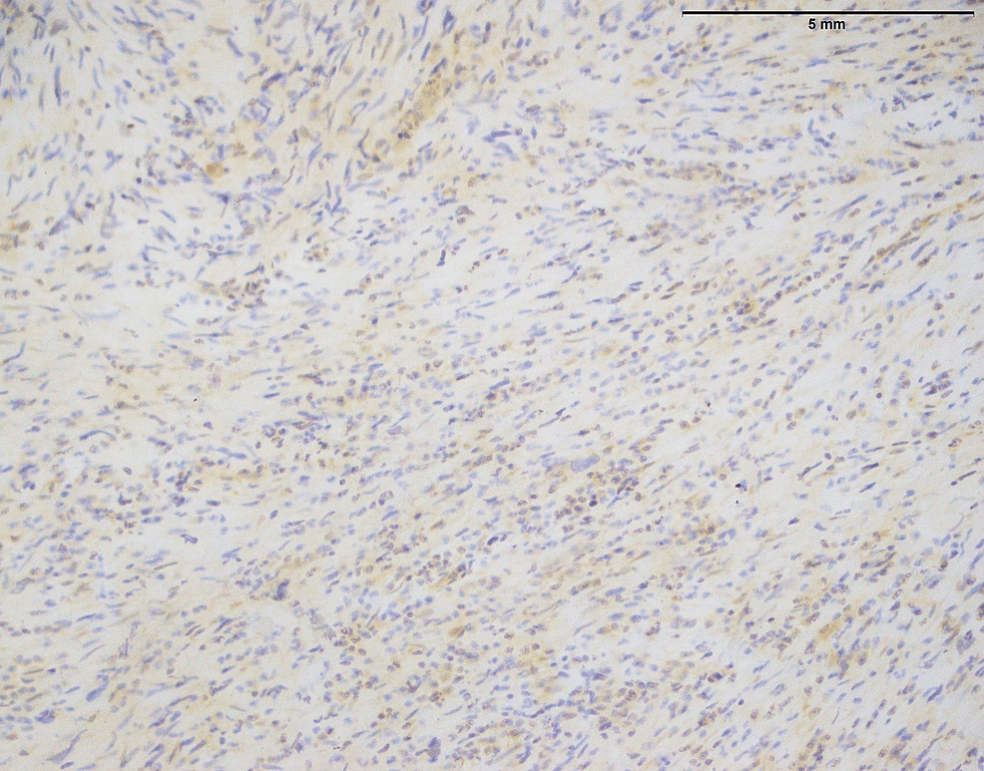
Liposarcoma of retroperitoneum showing positive expression for MDM2 (20x) MDM2: Murine double minute 2

**Figure 5:**
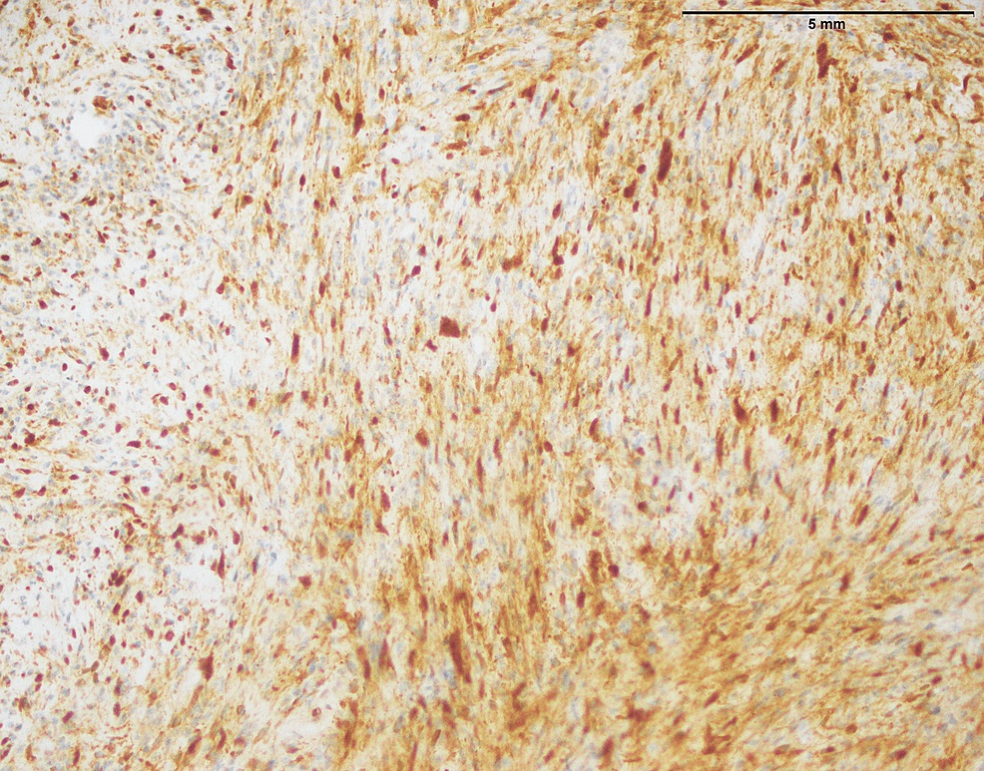
Liposarcoma of retroperitoneum showing positive expression for p16 (40x) p16 (also known as p16^INK4a^, cyclin-dependent kinase inhibitor 2A, CKN2A)

**Figure 6:**
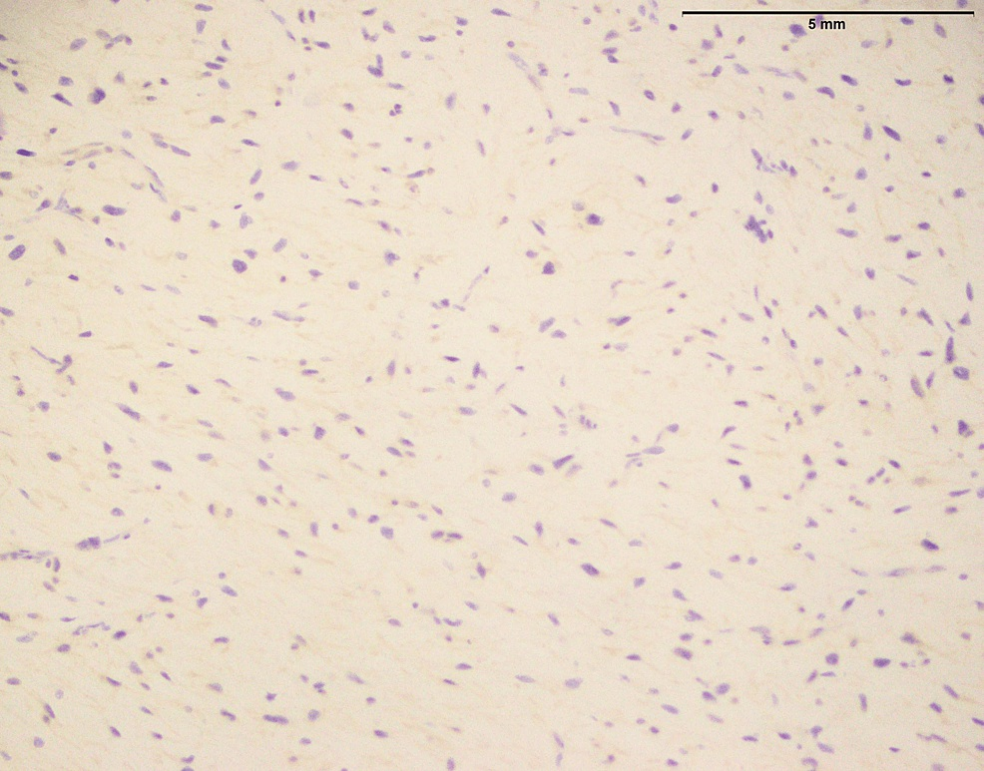
No expression of MUC4 in reteroperitoneal liposarcoma (40x) MUC4: Mucin-4

Liposarcoma is a form of malignant neoplasm that arises in adipose tissue. Twenty-three liposarcoma cases were studied, including 16 males and 7 females. The age range was from 11 to 70 years with mean and median ages were 43 and 44, respectively. The most common anatomical site was the lower limb (n=7) followed by the upper limb (n=5), retroperitoneum (n=4), back (n=2), abdominal wall (n=2), axilla (n=1), breast (n=1), and paraphryngeal space (n =1). Liposarcoma histopathology revealed a unique myxoid background, as shown in Figure 3. This myxoid backdrop refers to the presence of gel-like or mucinous material within the tissue (Figure 3). It produced a distinct appearance in microscopic examination, characterized by a transparent or mucoid appearance around the cells. The myxoid backdrop is a distinguishing characteristic of certain liposarcoma subtypes, such as myxoid liposarcoma. The tumor was made up of sheets of lipoblasts embedded in a myxoid backdrop. Lipoblasts are specialized cells containing a cytoplasmic lipid (fat) vacuole or droplet. Lipoblasts are distinguished by the presence of lipid droplets, which provide them the appearance of signet ring-like cells. Lipoblasts are characteristic of liposarcoma that distinguishes it from other soft tissue tumors. Giant cells, in addition to lipoblast, may be seen in tumor tissue. The nuclei of giant cells are excessively large. Their presence can indicate a more aggressive type of liposarcoma, and their detection is crucial in determining the tumor’s grade and tendency for metastasis. MDM2, which stands for mouse double minutes 2, was expressed in the liposarcoma tissue in these cases (Figure 4), MDM2 is a protein that is typically overexpressed in liposarcoma. Positive p16 expression indicates that tumor cells in this case of liposarcoma express the p16 protein (Figure 5). The absence of MUC4 expression (Table 1) in liposarcoma showed that this particular marker is not a dominant characteristic of this tumor type (Figure 6), which can assist in identifying it from other malignancies that may express MUC4.

**Figure 7:**
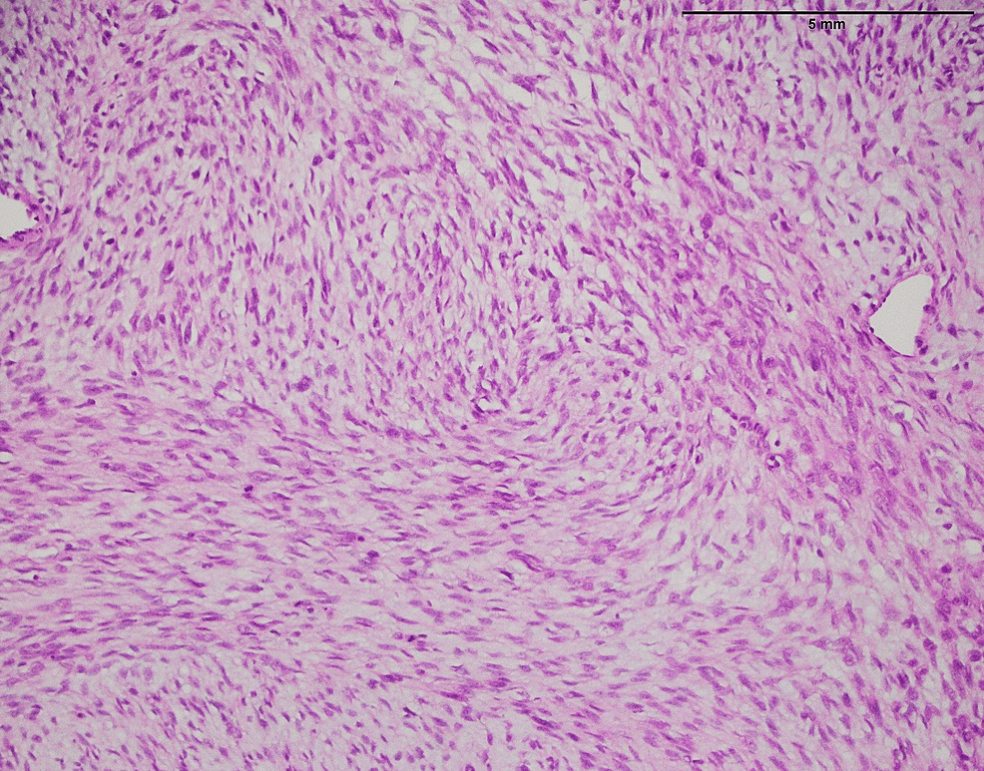
Malignant peripheral nerve sheath tumor of the calf: an infiltrative tumor composed of spindle cells with pleomorphic nuclei arranged in whorling and diffuse patterns (40x)

Distinct histopathological characteristics distinguish MPNST, as shown in Figure 7. Ten cases of MPNST including five males and five females were studied. The age ranged from 18 to 45 years with mean and median ages were 32 and 29 respectively. The common anatomical site was the lower limb (n=8) and the upper limb (n=2). During the examination, an infiltrative growth pattern was shown, indicating that the tumor can invade adjacent tissues (Figure 7). The tumor was made up of spindloid cells, which were elongated and spindly in form. The nuclei within these cells were pleomorphic, which means they fluctuate in size and form, indicating cellular atypia. These pleomorphic nuclei are notable for their unique patterns, which include whirling (spiral-like) and diffuse (disorganized) forms.

**Figure 8:**
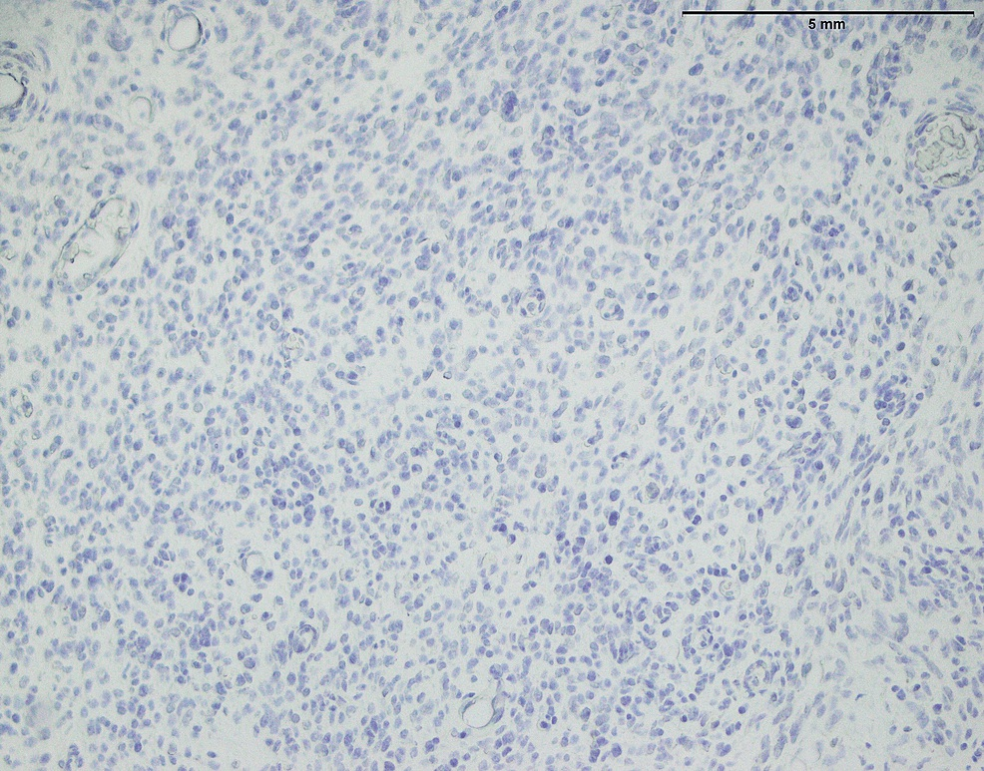
No expression of MUC4 in MPNST of lower limb (calf) (40x) MUC4: Mucin-4, MPNST: Malignant peripheral nerve sheath tumor

**Figure 9:**
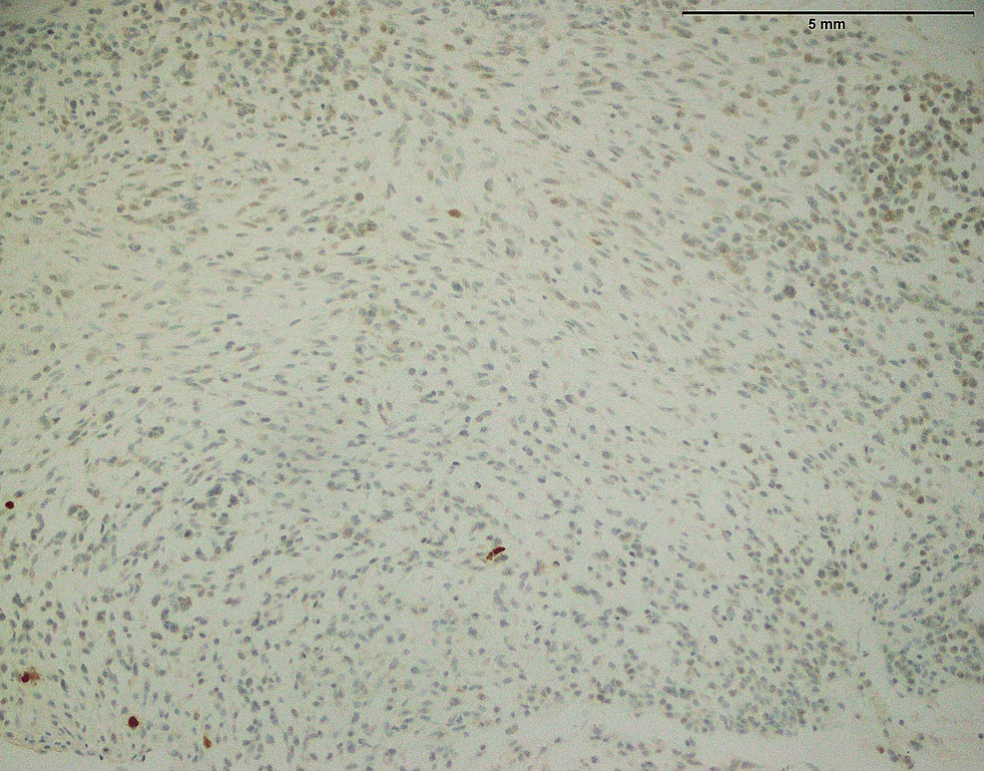
MPNST of the lower limb, showing positive expression of S-100 protein (40x) MPNST: Malignant peripheral nerve sheath tumor S-100 protein (solubility in 100%-saturated solution with ammonium sulphate at neutral pH)

MUC4 was not expressed in MPNST as shown in Figure 8. The lack of MUC4 expression in this setting showed that it is not a key marker for MPNST (Table 1). At the same time, A positive expression of S-100 protein was found in this instance of MPNST (Figure 9). S-100 protein is a marker usually linked with neural tissue and cells, and its presence in MPNST is noteworthy. This positive expression supports the tumor’s neural origin, which aids in diagnosis.

**Figure 10:**
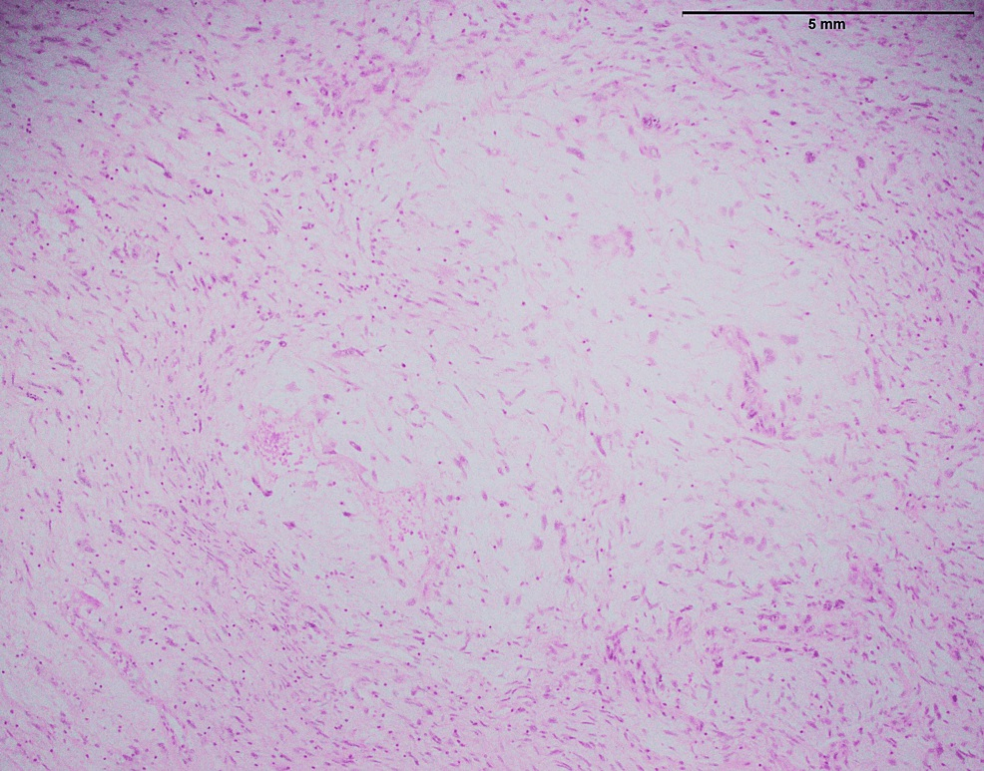
Myxofibrosarcoma involving chest wall: an infiltrative tumor arranged in a lobular configuration having fibromyxoid stroma and arcuate-like vascular pattern (40x)

**Figure 11:**
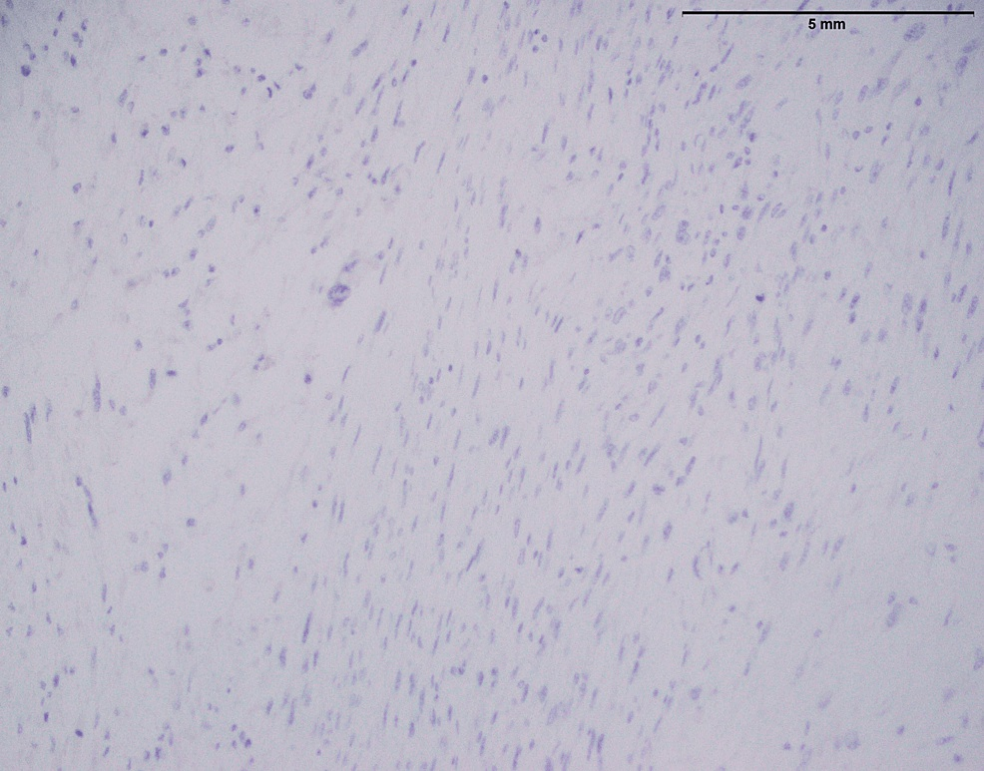
No expression of MUC4 in myxofibrosarcoma of the chest wall (40x) MUC4: Mucin-4

Twenty-six myxofibrosarcoma cases affected 21 males and 5 females with the age range of 24-80 years. The mean and median ages were 52 and 55, respectively. The common anatomical site of this sarcoma was the lower limb (n=12) followed by the upper limb (n=7), chest wall (n=4), back (n=2), and abdominal wall (n=1). Histopathological characteristics of myxofibrosarcoma are shown in Figure 10. Initially, it appeared as an infiltrative tumor, indicating a propensity to invade adjacent tissues. The tumor had a lobular shape, with cells clustered in rounded or lobulated clusters (Figure 10). It has a fibromyxoid stroma, which indicates a mixture of fibrous and mucinous material inside the tumor tissue. In addition, an arcuate-like vascular pattern was detected, indicating the existence of curved or arched-shaped blood vessels within the tumor. These characteristics jointly identify myxofibrosarcoma and help in its diagnosis. Similar to other sarcomas as shown in Table 1, there was no MUC4 expression in myxofibrosarcoma (Figure 11). The lack of MUC4 expression in this environment shows that it is not a significant marker for myxofibrosarcoma.

**Figure 12:**
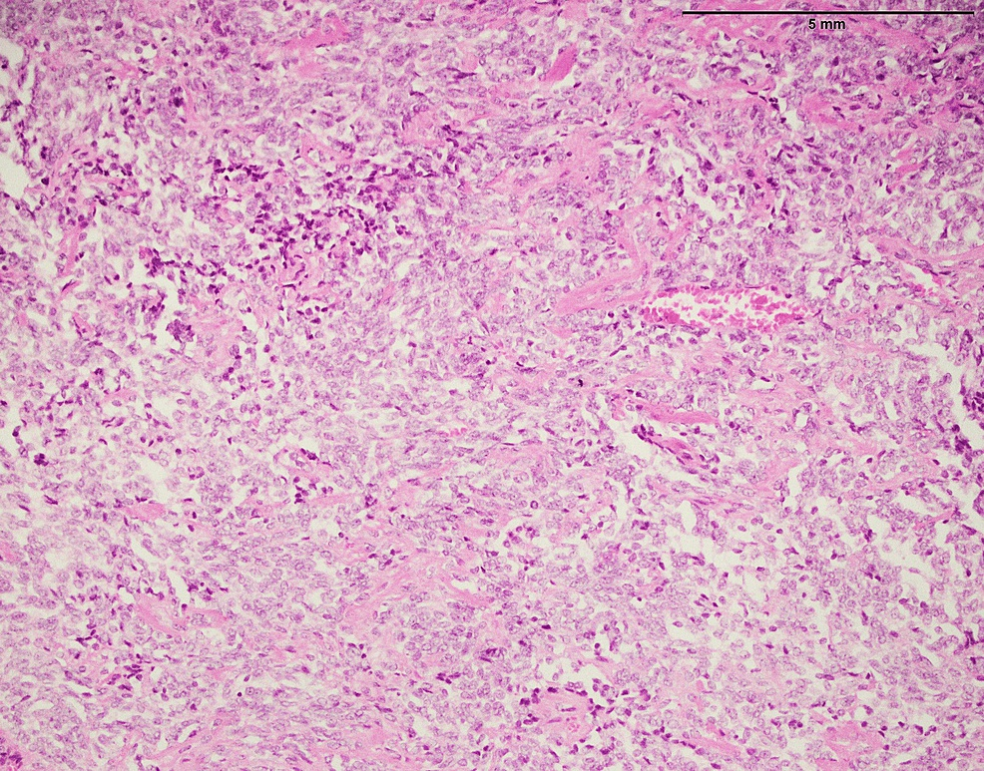
Synovial sarcoma of the forearm: an infiltrative tumor composed of spindle cells arranged in fascicles and background showing hemangiopericytoma-like vascular pattern (40x)

Synovial sarcoma as shown in Figure 12 appears as an infiltrative tumor that demonstrated a propensity to infiltrate neighboring tissues. A total of 13 synovial sarcoma cases, nine males and four females were studied. The age range of patients was 14-55 years with mean and median ages were 31 and 36 respectively. The common anatomical site was the lower limb (n=5) followed by the upper limb (n=4), abdominal wall (n=2), back (n=1), and chest wall (n=1). This tumor is mostly made up of elongated spindle cells that are grouped in fascicles, structured bundles, or groupings. The surrounding area of the tumor demonstrated a hemangiopericytoma-like vascular pattern, which was notable. This vascular pattern was similar to hemangiopericytoma, with branching and irregular blood vessels surrounded by spindle-shaped pericytic cells. These combination histological findings are characteristic of synovial sarcoma and are critical for correct diagnosis. Understanding these features helps to identify synovial sarcoma from other soft tissue cancers and discloses treatment methods for afflicted patients.

**Figure 13:**
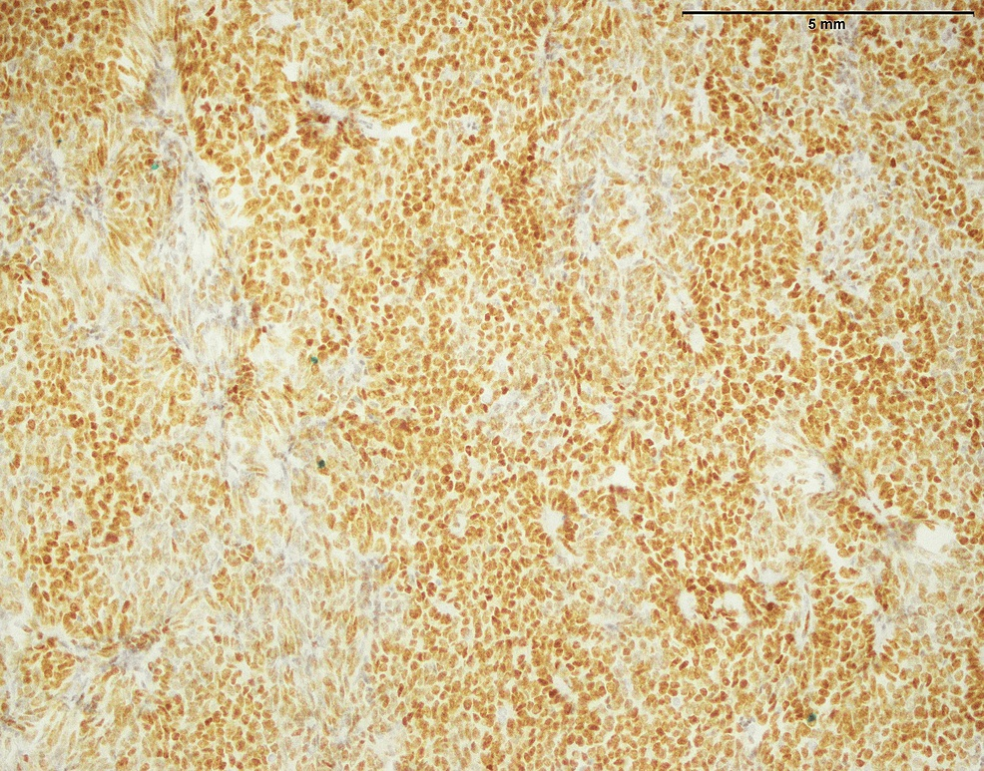
Upper limb synovial sarcoma showing strong positive expression of TLE1 (40x) TLE1: Transducin-like enhancer protein 1

**Figure 14:**
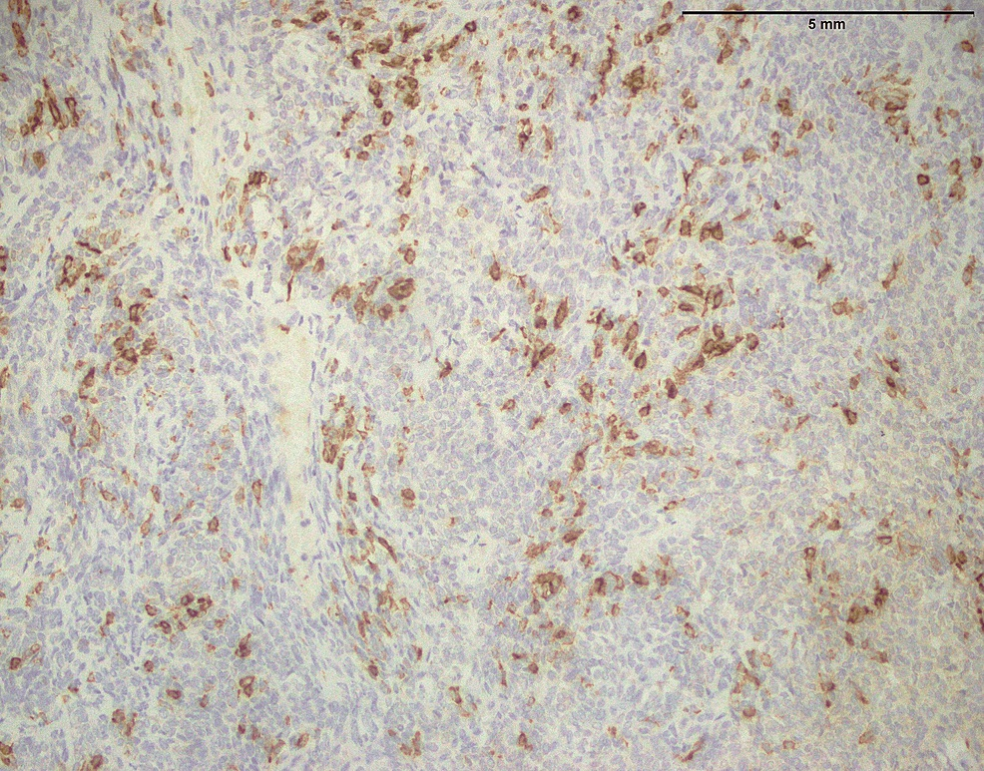
Upper limb synovial sarcoma showing focal positivity for MUC4 (40x) MUC4: Mucin-4

Two significant IHC findings were obtained in synovial sarcoma. TLE1, a recognized marker linked with synovial sarcoma, was found to be expressed positively (Figure 13). TLE1-positivity aids in the diagnosis of synovial sarcoma since it is a specific marker for this cancer. Second, there was focal positivity for MUC4 (Figure 14) in the n=1 case. While MUC4 is not usually thought of as a key marker for synovial sarcoma, localized positivity implies that it is expressed in certain instances (Table 1). The findings emphasize the importance of considering multiple makers when making a diagnosis. Overall, the combined IHC profile with TLE1 positivity provided useful diagnostic information and aids in the proper diagnosis of synovial sarcoma.

**Figure 15:**
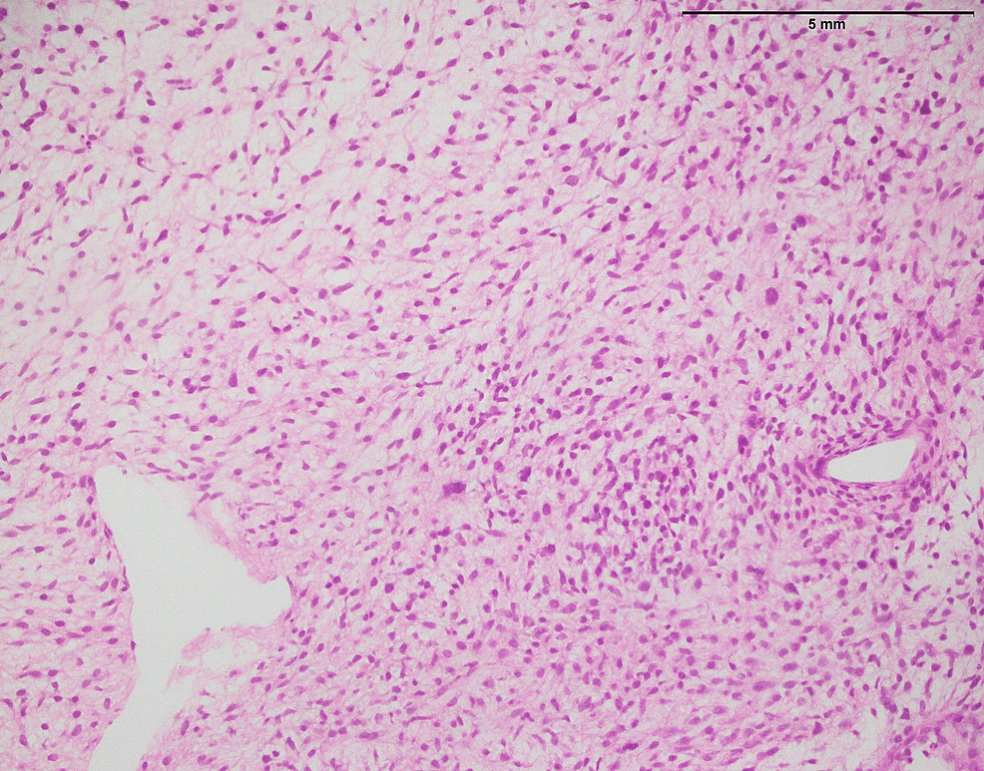
Leiomyosarcoma of lower limb; fascicles of smooth muscle fibers displaying a moderate degree of nuclear atypia and pleomorphism (40x)

**Figure 16:**
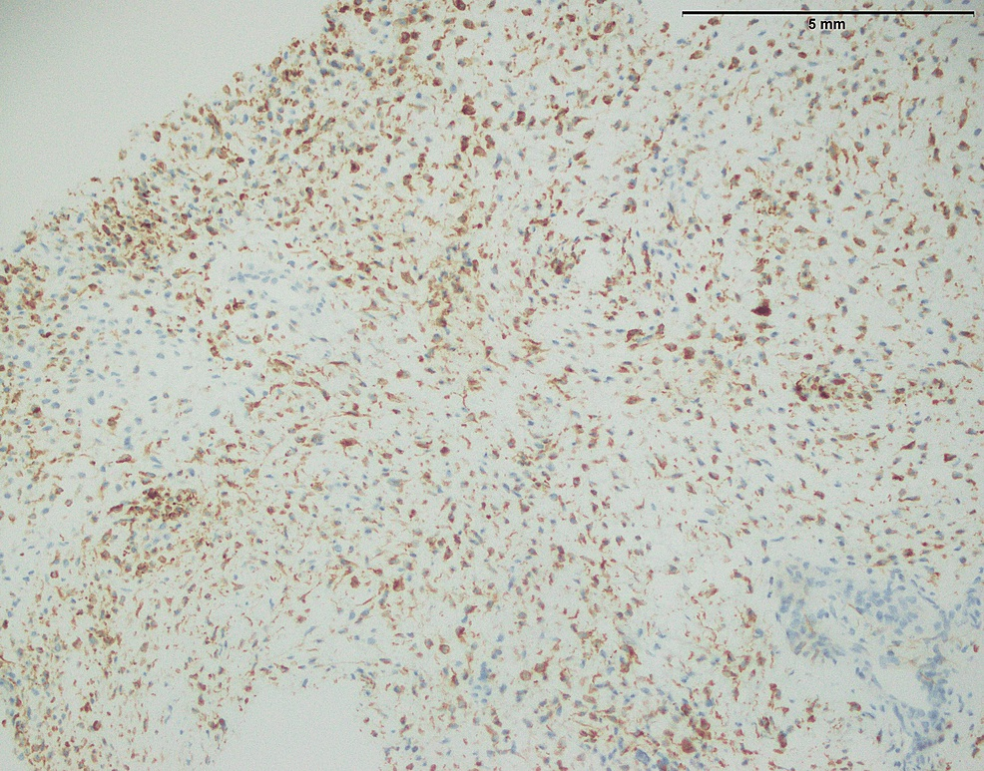
Leiomyosarcoma showing positive expression of desmin in smooth muscle fibers (40x)

**Figure 17:**
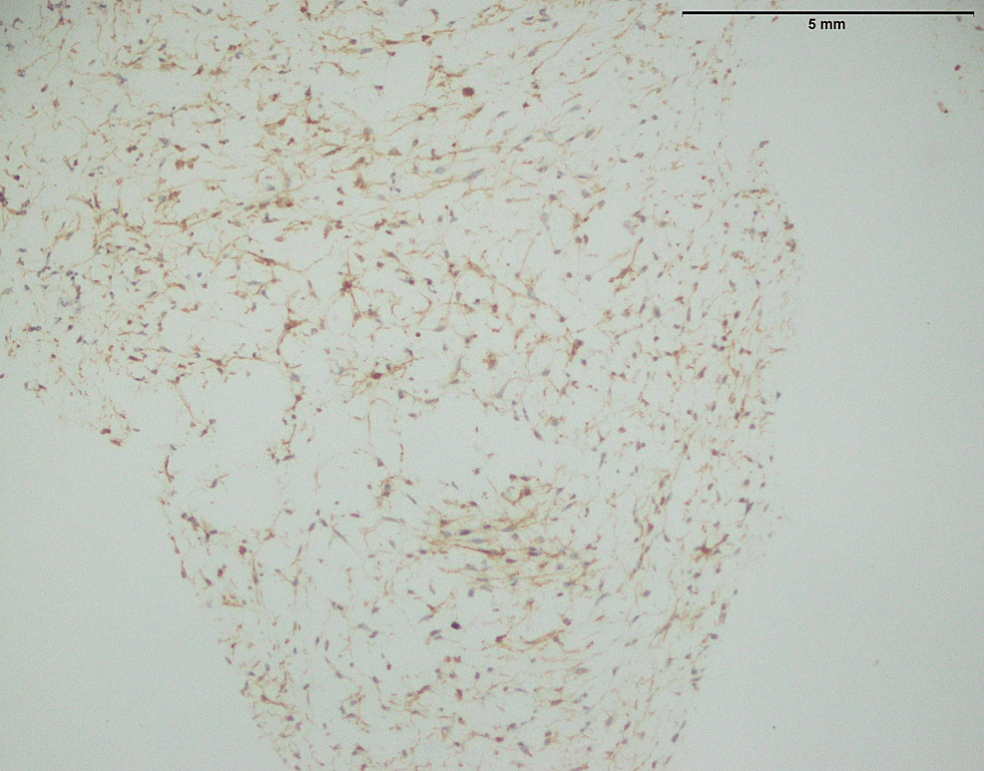
Leiomyosarcoma: Positive expression for smooth muscle fibers for Caldesmon (10x)

**Figure 18:**
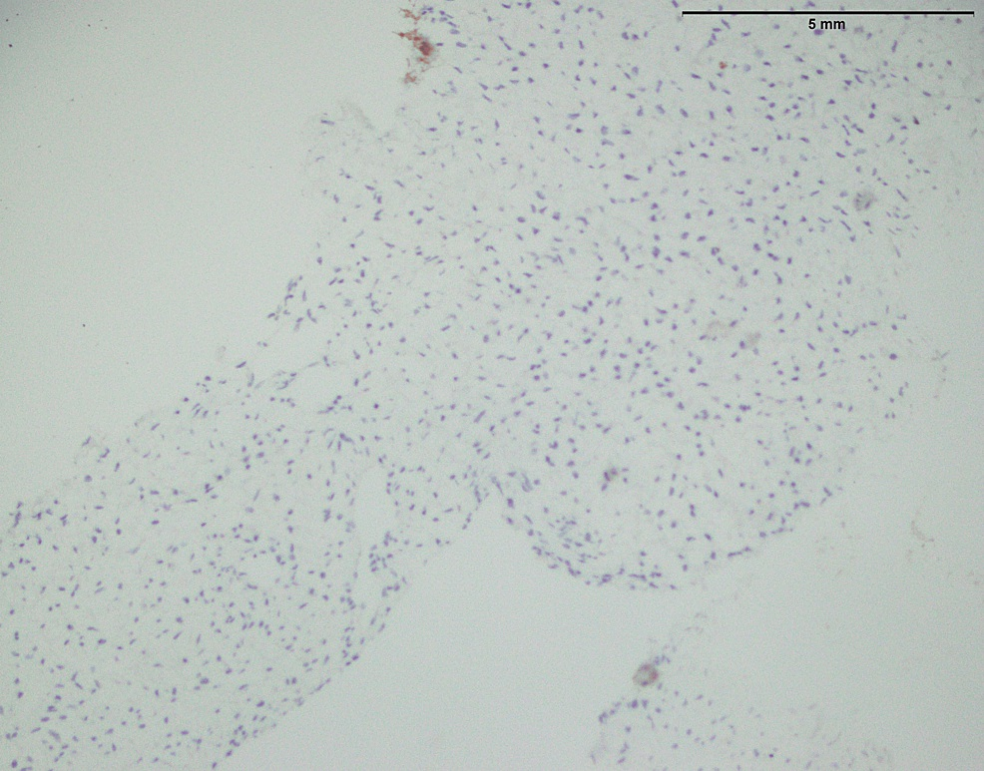
Leiomyosarcoma: No expression of MUC4 in the smooth muscle fibers (10x) MUC4: Mucin-4

Fifteen leiomyosarcoma cases reported in this study were affected (five males and ten females). The age range from 30 to 59 years with mean and median ages were 49 and 49 respectively. The most common anatomical site was the lower limb (n=8) followed by the upper limb (n=5), and chest wall (n=2). The photomicrograph revealed specific histopathological features in a tissue sample (Figure 15). It displayed fascicles of smooth muscle fibers, which exhibited moderate nuclear atypia and pleomorphism. This indicates that the muscle fibers had irregularities in terms of the size, shape, and appearance of their nuclei. Furthermore, the IHC staining results showed positive expression for smooth muscle fibers in two different markers: Desmin and Caldesmon which are proteins associated with smooth muscle tissue, and their positive expression confirms the presence of smooth muscle cells in the sample (Figure 16 and Figure 17). However, the IHC stain for MUC4 displayed no expression in the smooth muscle fibers (Table 1 and Figure 18).

Sixteen SEF cases with ten males and six females and a total of 14 LGFMS cases with eight males and six females were studied. In SEF, the age ranges from 14 to 55 years with mean and median ages were 35 and 36, respectively. In contrast, in LGFMS, the age ranges from 13 to 52 years with mean and median ages were 30 and 36, respectively. In SEF, the most common anatomical site was the lower limb (n=6) followed by the upper limb (n=3), trunk (n=2), and neck (n=2). While in LGFMS, the most common anatomical site was the lower limb (n=7) followed by the upper limb (n=4), trunk (n=2), and inguinal area(n=1).

**Figure 19:**
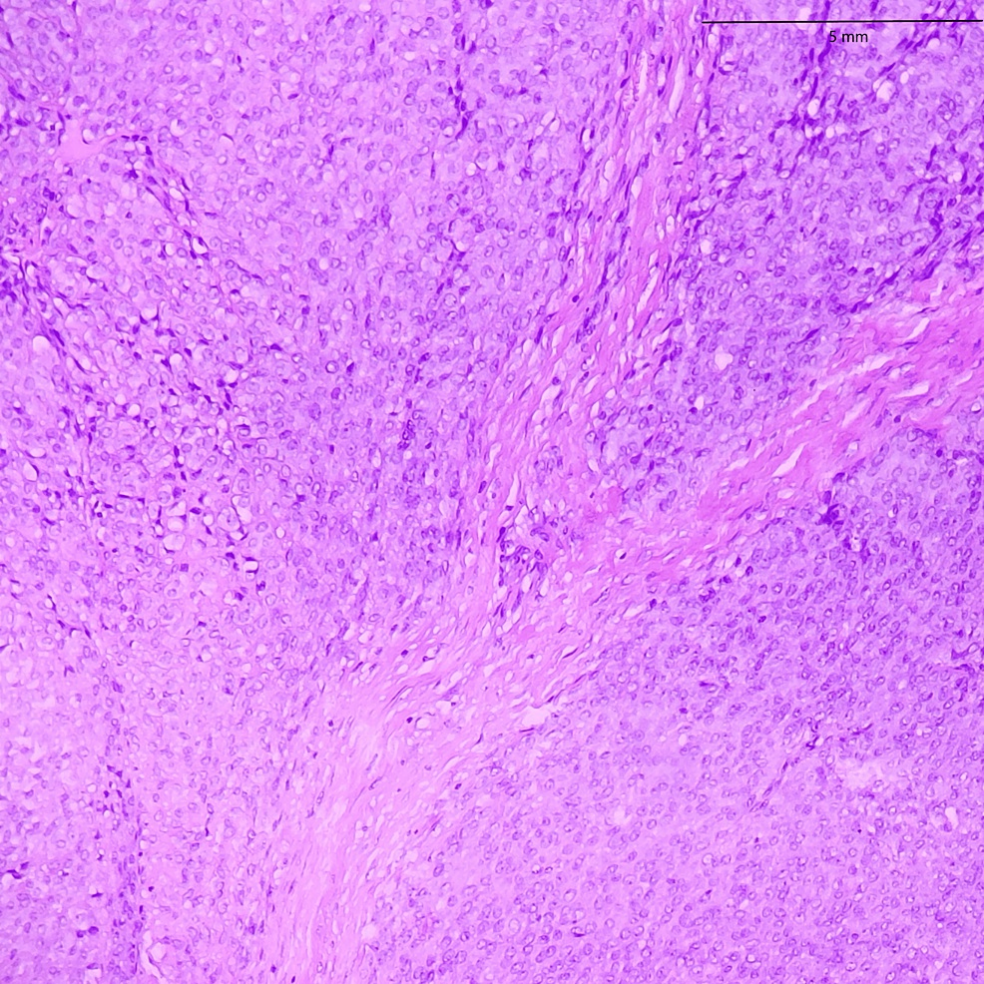
Sclerosing epithelioid fibrosarcoma involving the trunk, composed of round to polygonal epithelioid cells with sparse cytoplasm, round-to-oval nuclei, and inconspicuous nucleoli (20x)

**Figure 20:**
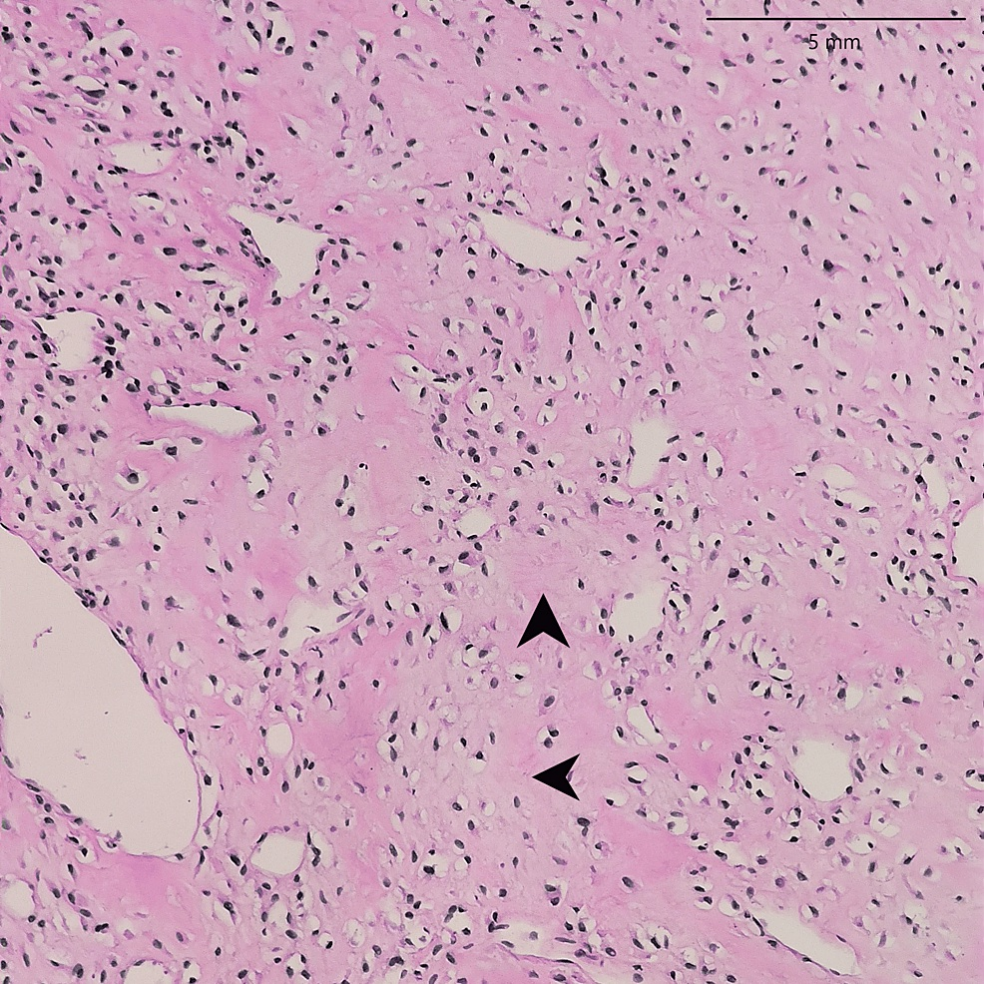
Low-grade fibromyxoid sarcoma: An infiltrative tumor of the upper limb showing moderately cellular bland-looking fusiform spindle cell with abrupt transition to myxoid areas (arrowhead) (40x)

SEF is primarily composed of round-to-oval epithelioid cells with limited cytoplasm, round-to-oval nuclei, and inconspicuous nucleoli (Figure 19). These cellular characteristics are typical of epithelioid cells. SEF is known for its unique epithelioid appearance and often displays a lack of spindle-shaped cells. This feature aids in its recognition and diagnosis. In contrast, LGFMS features moderately cellular, bland-looking fusiform spindle cells (Figure 20). A notable aspect is the abrupt transition to myxoid areas within the tumor. This abrupt transition is characteristic of LGFMS and distinguishes it from other soft tissue tumors.

**Figure 21:**
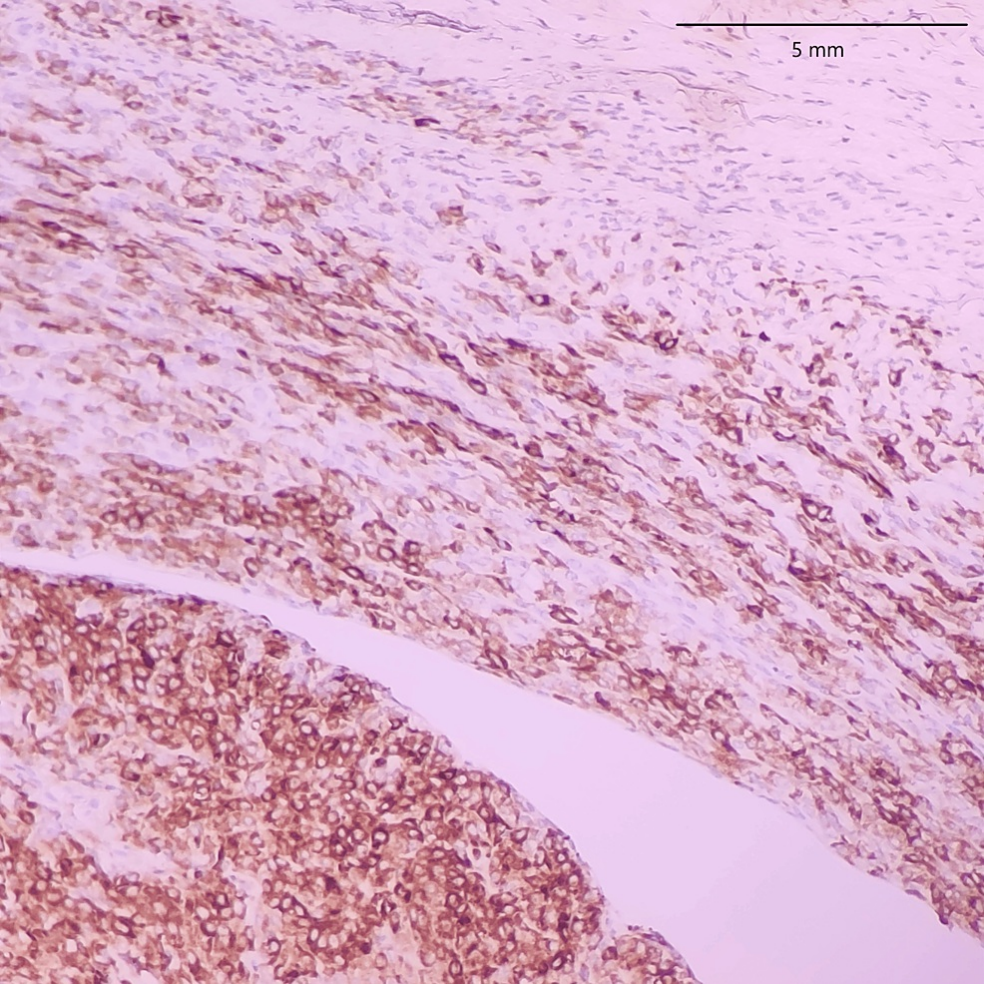
Positive expression of MUC4 in SEF of the trunk (20x) MUC4: Mucin-4, SEF: Sclerosing epithelioid fibrosarcoma

**Figure 22:**
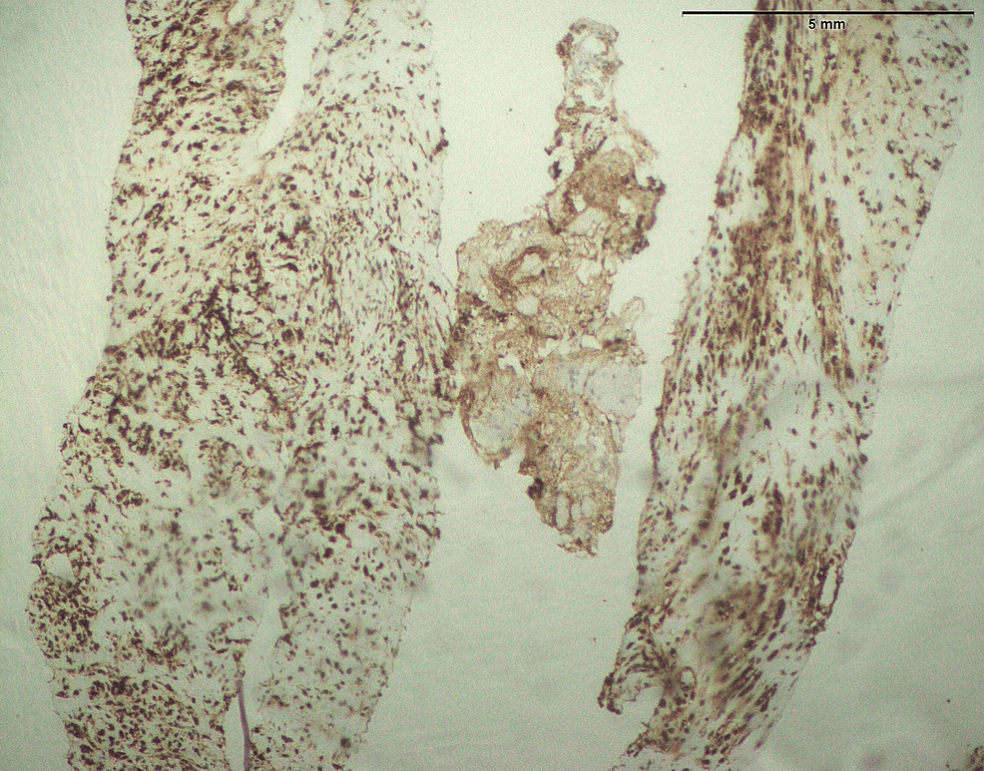
Strong positive expression of MUC4 in LGFMS (40x) MUC4: Mucin-4, LGFMS: Low-grade fibromyxoid sarcoma

In both SFE and LGFMS, there was a positive expression of MUC4, a glycoprotein marker (Figure 21 and Figure 22). The presence of MUC4 in both tumor types may hold diagnostic significance (Table 1), potentially highlighting a previously uncharacterized molecular aspect of these malignancies. This result underscores the importance of considering a wide range of molecular markers in the histopathological evaluation of sarcomas to aid in their accurate diagnosis and classification.

## Discussion

The study provides crucial insights into the diagnostic significance of MUC4, particularly in the context of specific sarcoma subtypes. This research not only provides crucial insights into these specific tumors but also highlights the broader context of histopathological and IHC characteristics in soft tissue sarcomas.

In the findings of the current study, SEF and LGFMS exhibit strong MUC4 expression (Figures 20, 22), other sarcoma types including fibrosarcoma (Figure 2), liposarcoma (Figure 6), MPNST (Figure 9), myxofibrosarcoma (Figure 11), and leiomyosarcoma (Figure 18) showed no MUC4 expression. The findings in this study align with Nagona et al. indicating that MUC4 strongly expresses in SEF and LGFMS [9,10]. These results reinforce the specificity of MUC4 as a reliable diagnostic marker for SEF and LGFS, as it consistently shows positive expression in these two particular sarcoma types.

The histopathological examination of fibrosarcoma cases (Figure 1) in this study reaffirms the classic characteristics associated with this malignancy, including an infiltrative growth pattern and the presence of spindle cells with varying degrees of atypia. In a previous study [11], histological aspects of fibrosarcoma studied and documented the presence of infiltrative growth patterns, as well as spindle cells with varying degrees of atypia, as hallmark features of this malignancy. However, it is essential to emphasize that the study's primary focus on MUC4 expression in specific sarcomas, including SEF and LGFS, adds depth to our understanding of sarcoma markers.

Liposarcoma with histopathological characteristics (Figure 3) such as a myxoid background and lipoblasts corresponds to previously reported findings regarding this. A previous study has reported that a myxoid stroma and the presence of lipoblasts are important diagnostic factors for a particular type of cancer [12]. The presence of MDM2 and p16, both recognized liposarcoma markers, adds to their diagnostic usefulness (Figures 4, 5). Importantly, the absence of considerable MUC4 expression in liposarcoma distinguishes it from other types of sarcomas. The histopathological investigation of MPNST in this work confirms well-known features of these aggressive neoplasms (Figure 7). Previous studies have consistently shown an infiltrative growth pattern and the presence of pleomorphic nuclei, which are generally symptomatic of high-grade malignancy [13]. These characteristics highlight the aggressive nature of MPNSTs and the importance of early diagnosis. Furthermore, the presence of S-100 protein in MPNSTs (Figure 9) is a key diagnostic indication since it indicates their neuronal origin. These findings correlate with the findings of Maria et al. that highlighted the importance of S-100 protein expression in differentiating MPNSTs from other soft tissues [14,15].

The present study adds to our understanding of myxofibrosarcoma by thoroughly characterizing its histological characteristics (Figure 10), such as its infiltrative nature, lobular form, fibromyxoid stroma, and arcuate-like vascular pattern. Washimi et al. emphasized the infiltrative growth pattern and fibromyxoid stroma in their investigations [16], whereas Stephen et al. emphasized the lobular form and characteristic arcuate-like vascular pattern [17]. These consistent findings across investigations improve our collective understanding of the histological characteristics of myxofibrosarcoma. Myxofibrosarcoma’s IHC profile is influenced by the lack of substantial MUC4 expression (Figure 11). Furthermore, the existence of smooth muscle fibers in leiomyosarcoma cases was shown by histological results, which demonstrated mild nuclear atypia and pleomorphism, indicating nuclei abnormalities. IHC staining for Desmin and Caldesmon, two well-known smooth muscle tissue markers, showed the expression in leiomyosarcoma cases (Figures 16, 17) [18], which was consistent with prior research conducted by Hasegawa et al. and Robin et al. [19]. Notably, the IHC stain for MUC4 showed no expression, in line with previous research findings on the limited presence of MUC4 in smooth muscle tissue [20].

However, synovial sarcoma, sharing key histopathological features with other sarcomas, displays an infiltrative growth pattern and spindle cell morphology (Figure 12). However, its unique hemangiopericytoma-like vascular pattern sets it apart. The histopathological features of synovial sarcoma, particularly its infiltrative growth pattern and spindle cell morphology, have been well-documented in previous studies [21,22]. What distinguishes synovial sarcoma is its characteristic hemangiopericytoma-like vascular pattern, as highlighted by Fiore et al. [23] and further supported by Lan et al. work [24]. These distinguishing characteristics aid in the knowledge of synovial sarcoma pathophysiology and distinction from other sarcomas. The positive expression of TLE1, a particular marker (Figure 13), validates the diagnosis, while the focal positivity for MUC4 (Figure 14) underlines the significance of examining numerous markers.

Gender and age variations in MUC4 expression in SEF, LGFS, and many other conditions have not been substantially studied in the literature. Most investigations concentrate on MUC4’s diagnostic usefulness rather than its relationship to gender. As a result, the effect of gender and age on MUC4 expression in these sarcomas was studied in the current study, but the findings supported the previously published literature.

Recognizing MUC4 as a sensitive and very effective marker in these circumstances provides pathologists and physicians with a vital tool [4,5]. However, it is critical to point out that the overall sarcoma landscape is diverse. The findings show that there is no considerable MUC4 expression in other sarcoma types, emphasizing the importance of broad diagnostic panels. These panels should include a variety of makers to ensure accurate diagnosis, as seen by the presence of TLE1 in synovial sarcoma (Figure 13) or MDM2 in liposarcoma (Figure 4) [25,26]. Future research should look at the molecular process underpinning marker expression patterns and their implications for therapy.

## Conclusions

In conclusion, the results of our research emphasize the diagnostic importance of MUC4 in these particular sarcoma subtypes. This study highlights the diagnostic significance of MUC4, notably in LGFS and SEF. These findings have obvious implications for the correct identification and treatment of these difficult sarcoma subtypes.

These findings provide important recommendations for physicians and pathologists while also showing the complexity and variability of the sarcoma landscape. More research and comprehensive diagnostic techniques are needed to advance our knowledge and management of these uncommon soft tissues. Further research is needed to understand the molecular mechanism that governs marker expression patterns, as well as the therapeutic implications.
